# Effect of Glycated Hemoglobin (HbA1c) and Duration of Disease on Lung Functions in Type 2 Diabetic Patients

**DOI:** 10.3390/ijerph18136970

**Published:** 2021-06-29

**Authors:** Hawra Bin Maan, Sultan Ayoub Meo, Fawziah Al Rouq, Imran Muhammad Umar Meo, Milagros E. Gacuan, Joud Mohammed Alkhalifah

**Affiliations:** Department of Physiology, College of Medicine, King Saud University, Riyadh 11461, Saudi Arabia; 437202999@ksu.edu.sa (H.B.M.); falrouq@ksu.edu.sa (F.A.R.); Imeo@ksu.edu.sa (I.M.U.M.); milgacuan@ksu.edu.sa (M.E.G.); 438202353@ksu.edu.sa (J.M.A.)

**Keywords:** diabetes mellitus, glycated hemoglobin, HbA1c, lung functions

## Abstract

Diabetes mellitus is a highly challenging global health care problem. This study aimed to assess the effect of glycated hemoglobin (HbA1c) and duration of diabetes on lung function in type 2 diabetic patients and assess whether duration or high HbA1c is more noxious to damage the lung functions. A total of 202 participants, 101 patients with type 2 diabetes mellitus (T2DM), and 101 age-, gender-, height-, and weight-matched controlled subjects were recruited. The HbA1c was measured through a clover analyzer, and lung function test parameters were recorded by spirometry. The results revealed a significant inverse correlation between HbA1c and Vital Capacity (VC) (r = −0.221, *p* = 0.026), Forced Vital Capacity (FVC) (r = −0.261, *p* = 0.008), Forced Expiratory Volume in First Second (FEV1) (r = −0.272, *p* = 0.006), Forced Expiratory Flow 25% (FEF-25%) (r = −0.196, *p* = 0.050), Forced Expiratory Flow 50% (FEF-50%) (r = −0.223, *p* = 0.025), and Forced Expiratory Flow 75% (FEF-75%) (r = −0.169, *p* = 0.016). Moreover, FEV1 (*p* = 0.029), FEV1/FVC% (*p* = 0.006), FEF-50% (*p* = 0.001), and FEF-75% (*p* = 0.003) were significantly lower in the diabetic group with duration of disease 5–10 and >10 years compared to the control group. The overall results concluded that high HbA1c or uncontrolled diabetes mellitus has a more damaging effect on lung function impairment compared to the duration of diabetes mellitus. Physicians must regularly monitor the HbA1c level while treating diabetic patients, as good glycemic control is essential to minimize the complications of DM, including lung function impairment in patients with T2DM.

## 1. Introduction

Worldwide, diabetes mellitus (DM) has become a challenging pandemic due to population growth, aging [1], sedentary lifestyle, [2] obesity, unplanned urbanization, industrialization, and environmental pollution [3,4]. Diabetes mellitus affects all the organs in the human body and accounts for the economic burden among the patients and societies [5]. The prevalence of DM is 463 million; 374 million people suffer from impaired glucose tolerance, and 232 million people are unaware that they are suffering from the disease [5].

Uncontrolled diabetes mellitus and hyperglycemia contribute to poor outcomes in vascular diseases. Hyperglycemia and the duration of the disease are major risk factors for the progress of various micro-angiopathic-associated complications [6]. In uncontrolled diabetes, dysglycemia increases reactive oxygen species, and variations in signaling pathways cause various vascular dysfunctions [7]. In addition, it may induce numerous complications in all organs, including the cardiorespiratory system [8]. 

The lung is one of the targeted body organs in diabetes mellitus, in which impairment is relatively subclinical and often ignored by patients and physicians. However, the respiratory system is the most vulnerable human body system, and the lung is susceptible to diabetic microvascular complications [9]. In addition, the earlier literature demonstrated that biochemical and micro-angiopathic alterations of elastic recoil properties cause lung damage in the diabetic population [9,10].

The literature has shown that the length of disease is the primary cause of various complications of diabetes mellitus [11]. Interestingly, there is debate in the scientific community that the commencement and development of vascular complications are mainly due to the duration of diabetes, but uncontrolled diabetes is also involved in lung impairment. However, it is not clear which one is more toxic, the duration of diabetes mellitus or high hyperglycemic condition due to increased glycated hemoglobin (HbA1c). Therefore, the present study aimed to investigate the effect of diabetes and HbA1c on lung function impairment in T2DM patients and to understand which one is more toxic, either the duration of diabetes mellitus or uncontrolled diabetes mellitus due to increased HbA1c. 

## 2. Subjects ad Methods

The present matched case-controlled cross-sectional study was conducted in the “Department of Physiology, Clinical Physiology Unit, College of Medicine, King Abdulaziz University Hospital, King Saud University, Riyadh, Saudi Arabia”. We recruited male and female subjects with type 2 diabetes mellitus, age range between 30–65 years, body mass index (BMI) less than 30 kg/m^2^, fasting blood glucose ≥126 mg/dl or HbA1c > 6.5%. The control group was free from any chronic diseases such as diabetes mellitus, hypertension, dyslipidemia, and cardiorespiratory conditions. 

### 2.1. Exclusion Criteria

Subjects with BMI > 30 kg/m^2^, cigarette smokers, and diabetic patients with a known history of coronary artery disease, retinopathy, neuropathy, nephropathy, cerebrovascular accident, type 1 diabetic patients, abnormality in vertebral column or thoracic cage, cancer, and who underwent thoracoabdominal surgery were excluded from the study [12,13].

Based on the inclusion and exclusion criteria, 101 type-2 diabetic patients were recruited. The diabetic subjects were matched for age, height, weight, BMI, ethnicity, and socioeconomic status with 101 control subjects. The healthy control subjects were selected from the university staff, including technicians and clerical staff. There were no significant statistical differences in the anthropometric means between the groups (Table 1). 

### 2.2. Measurements of HbA1c

HbA1c was measured by using a device “Clover A1c system (Inforpia, Kyunggi, Korea), an automated boronate affinity assay for the determination of the percentage of HbA1c % in the blood” [14]. The Clover A1c system is well acknowledged in the measurement of HbA1c both in clinical medicine and research. In addition, HbA1c is a reliable indicator of glycemic measures for the diagnosis of diabetes mellitus [15].

### 2.3. Spirometry

An Electronic Spirometer (Schiller AT-2 Plus; Schiller AG, Baar, Switzerland) was used to perform spirometry. The ventilatory lung function test parameters were performed under the supervision of the research team, using the same equipment at a fixed time of day. All the subjects were instructed about the spirometry techniques; the procedure was explained, and subjects were asked to perform normal inhalation and exhalation three times, followed by inspiration as deep as possible and then to exhale as rapidly as possible. The best results were taken after implementing three repeated cycles after the proper time of rest in a standing position. This procedure was based on the “American Thoracic Society of Standardization. The ventilatory lung function test parameters Vital Capacity (VC), Forced Vital Capacity (FVC), Forced Expiratory Volume in First Second (FEV1), Forced Expiratory Ratio (FEV1/FVC), Forced Expiratory Flow (FEF-25%), Forced Expiratory Flow 50% (FEF-50%), Forced Expiratory Flow 75% (FEF-75%), Forced Expiratory Flow 25–75% (FEF-25–75%), and Peak expiratory flow (PEF) were recorded.

### 2.4. Statistical Analysis

The data were analyzed using the Statistical Package for Social Sciences (SPSS 26; IBM Corp., New York, NY, USA). The mean ± SD was reported for quantitative variables for lung function parameters. A two-independent sample *t*-test was used to compare the mean differences between diabetic and control groups with overall lung function test parameters and then in the stratified analysis, i.e., duration of diabetes (less than or up to 5 years, 5–10 years, and longer than ten years) and HbA1c (≤8 and >8). Pearson Correlation was also applied to observe the overall and stratified relationship of HbA1c and duration of disease with lung function parameters. Binary logistic regression analysis was also performed to observe the log-odds in patients compared to the controls in lung function parameters. A *p*-value less than 0.05 was considered statistically significant.

## 3. Results

### 3.1. Demographic and Biochemical Characteristics

A total of 202 participants, 101 Type 2 diabetic patients and 101 age-, gender-, height-, and weight-matched controlled subjects were recruited. The mean age of the diabetic patients was 55.50 ± 5.99 years, ranging from 30–65 years, and the mean age for the control group was 54.88 ± 6.76 years, ranging from 30–65 years (Table 1). Among patients, most were males, 71 (70.29%), compared with females, 30 (29.71%). The mean duration of disease was 17 ± 7.64 years, ranging from 1–30 years. Among 101 controls, most were males, 70 (69.30%) compared to the females, 31 (30.70%). No significant difference was observed between the age (*p* = 0.489), height (*p* = 0.919), weight (*p* = 0.653), and BMI (*p* = 0.319) of diabetics and controls (Table 1). HbA1c and fasting blood glucose were significantly raised (*p* < 0.001) in the diabetic group as compared to the control group (Table 1).

### 3.2. Pulmonary Function Test

The subjects with diabetes duration less than or up to five years accounted for 14% of the total diabetic group. The mean duration of the disease was 3.86 ± 1.35 years. When the pulmonary functions were compared between the diabetics and matched controls, the mean difference between the VC, FVC, FEV1, PEF, FEV1/FVC%, and FEF 25–75% was not significant (Table 2). 

### 3.3. Duration of Diabetes 5–10 Years (Pulmonary Function Tests)

The subjects with diabetes duration between 5–10 years accounted for 26% of the total diabetic group. The mean duration of disease was 9.0 ± 1.07 years. When the pulmonary functions were compared between the diabetics and matched controls, the mean difference between the VC, FVC, FEV1, PEF, and FEF-25 was not statistically significant (*p* > 0.05). However, the mean FEV1/FVC% and FEF-50%, and FEF-75% were significantly lower in the diabetic group as compared to the control group (*p* < 0.05) (Table 3).

### 3.4. Duration of Diabetes >10 Years and Pulmonary Function Tests 

The diabetic patients with diabetes duration longer than ten years accounted for 61% of the total diabetic group. The mean duration of disease was 20.89 ± 4.70 years. When the pulmonary functions were compared between the diabetics and matched controls, the mean difference between the VC, FVC, FEV1/FVC%, PEF, and PEF 25–75% was not statistically significant (*p* > 0.05). However, the mean FEV1 was significantly lower in the diabetic group than in the control group (*p* = 0.029) (Table 4). 

### 3.5. HbA1c < 8 and Pulmonary Function Tests 

There were 58 subjects with HbA1c ≤ 8 (57.42% of the total diabetic group). When the pulmonary functions were compared between the diabetics and matched controls, the mean difference between the VC, FVC, FEV1, PEF, FEF-25%, and PEF75% was not statistically significant (*p* > 0.05). However, the mean FEV1/FVC% (*p* = 0.006) and FEF 50% (*p* = 0.034) were significantly lower in the diabetic group compared to the control group (Table 5). 

### 3.6. HbA1c > 8 and Pulmonary Function Tests 

There were 43 diabetic patients with HbA1c > 8 (42.57% of the total diabetic group). When the lung function parameters were compared between diabetics and matched controls, the mean difference between the VC, FVC, FEV1, PEFR, and PEF-25–75% was significantly lower in the diabetic group compared to the controls (*p* < 0.05). However, the mean FEV1/FVC% (*p* = 0.175) was not statistically significant (Table 6). 

### 3.7. Correlation Analysis (HbA1c and Lung Function Parameters)

**Figure 1:**
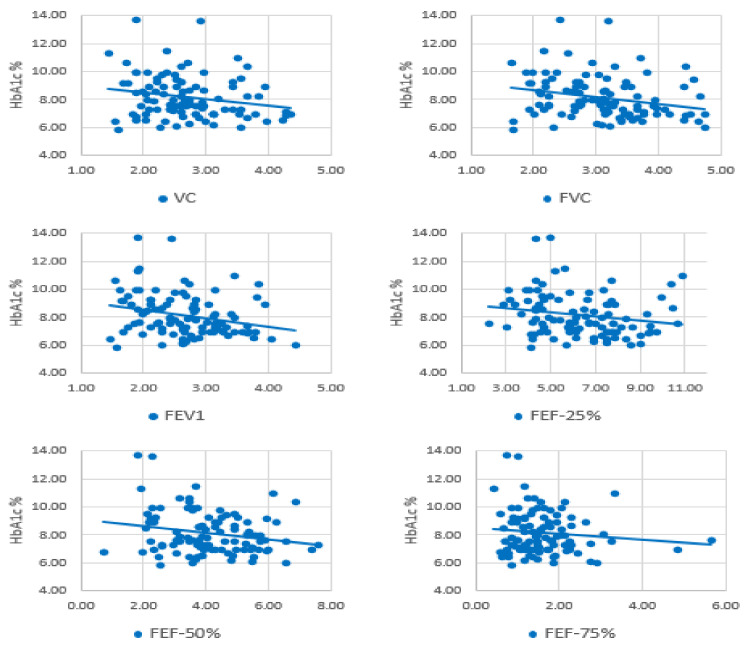
Correlation analysis between HbA1c and lung function parameters.

A significant inverse correlation was observed between HbA1c and VC (r = −0.221, *p* = 0.026), FVC (r = −0.261, *p* = 0.008), FEV1 (r = −0.272, *p* = 0.006), FEF-25% (r = −0.196, *p* = 0.050), FEF-50% (r = −0.223, *p* = 0.025), and FEF-75% (r = −0.169, *p* = 0.016) (Figure 1). However, relationships of HbA1c with FEV1/FVC (r = −0.013, *p* = 0.898) and PEF (r = −0.181, *p* = 0.070) were not significant.

### 3.8. Correlation Analysis (Duration of Diabetes and Lung Function Parameters)

There was no significant relationship observed between the duration of diabetes and lung function parameters (*p* > 0.05). Furthermore, logistic regression analysis of lung function parameters was performed, the results showed that VC, FVC, FEV1, PEF, and FEF 25–75% were not significantly decreased. 

## 4. Discussion 

Diabetes mellitus gradually affects all the body organs and systems with multi-systemic complications. Currently, diabetes mellitus has a high priority rank on the international health agenda due to being a global pandemic and a deathtrap to human health and worldwide economies [4]. Moreover, in the current global COVOID-19 pandemic, the diabetic population is at higher risk, as the infection involves the respiratory system [16]. The present study results demonstrated an association between the glycemic state and reduced lung function. Furthermore, we identified a significant inverse correlation between HbA1c and lung function parameters VC, FVC, FEV1, FEF-25%, FEF-50%, and FEF-50%. Thus, the overall result reveals that high HbA1c or uncontrolled diabetes mellitus impairs lung functions. 

Sushil and Mandira 2019 [17] conducted a study and reported that diabetic patients with a disease duration of longer than five years showed a significant reduction in ventilatory functions than control subjects. Mondal et al., 2019 [18] showed a substantial decline in FVC, FEV1, and PEFR in patients with T2DM compared to the control group. Zaman and Mallik, 2018 [19] conducted a study and found an insignificant deterioration in PFT parameters, FVC, FEV1, FEV1/FVC%, and PEFR, in diabetic patients older than 45 years. 

Meo and colleagues (2006) [20] reported that “diabetic patients had a significant reduction in the forced vital capacity (FVC), forced expiratory volume in one second (FEV1), and peak expiratory flow (PEF) relative to their matched controls. However, there was no significant difference in the forced expiratory ratio (FEV1/FVC%) and middle half of the FVC (FEF 25–75%) between the groups”. 

Díez-Manglano et al. 2021 [21] conducted a meta-analysis to hypothesize that the lung is a target organ of T2D. The authors identified a relationship between T2DM and impaired lung function, including FVC, FEV1, PEF, and FEF 25–75%, independent of gender, smoking habits, BMI, and geographical region. 

Pulmonary function parameters, FVC, FEV1, and FEF 25–75%, have significant changes in diabetic patients compared to healthy individuals [22,23]. The mean outcome of values of FVC, FEV1, PEFR, and FEF 25–75% was less in type 2 diabetes cases than in control subjects. The findings correlated with some other studies [24]. Similarly, in the present study, lung function parameters were decreased in T2D patients. The result reveals that high HbA1c or uncontrolled diabetes mellitus impairs lung functions. 

More recently, a study conducted by Tai et al., 2021 [25] found that pulmonary function indexes were negatively correlated with HbA1c and diabetes duration. Similarly, a study performed by Faisal et al., 2018 [26] showed a reduction in ventilatory functions compared to the control group; there was a negative correlation between raised HbA1c levels with reduced lung diffusion capacity predictive values. In another study, it was reported that diabetic patients with HbA1c equal to or higher than 8.0% had a 2.4-fold higher risk of lung function impairment than patients with HbA1c less than 6.9%. Thus, hyperglycemia is linked to lung function impairment. Similarly, the present study revealed a significant inverse relationship between HbA1c and VC, FVC, FEV1, FEF-25%, and FEF-50%.

Sonoda et al., 2018 [27] found that diabetic individuals with poor glycemic control had an elevated risk of lung function impairment. It has also been reported that diabetes is associated with reduced strength and endurance of respiratory muscles, particularly the diaphragm [28] and respiratory muscle mass [29]. It shows that poor glycemic control declines in respiratory muscles’ strength and endurance, leading to lung function impairment [28]. It has also been reported that DM substantially impaired respiratory muscles’ ultrastructure [30] and neuromuscular functions [31]. However, glycemic control can improve muscle mass and its associated physiology [32]. 

Continuously high blood glucose levels may alter the inflammatory pathways are involved in lung function impairment. Oxidative stress and non-enzymatic glycation of proteins are recognized in the etiology of diabetic lung injury [33]. Previously, the literature showed that elevated HbA1c was associated with decreased FVC and FEV1 [34]. Similarly, the present study regression analysis revealed that HbA1c was inversely related to FVC and FEV1 and other parameters in diabetic patients. 

There are some physiological and anatomical abnormalities in the lungs of diabetic patients that could explain the impaired lung function mechanism. The diabetic patients with poor glycemic control (HbA1c > 8) had significantly reduced lung functions compared to the duration of diabetes mellitus. A high HbA1c level is associated with reduced lung functions and is a reliable predictor of poor lung function. Hyperglycemia gradually damages the respiratory system by microangiopathy that leads to pulmonary injury [35]. The mechanism underlying these changes due to increased glucose level in the cell leads to conversion of glucose into sorbitol by aldose reductase and induces cell death through osmotic pressure. It can also be due to the induction of oxidative stresses, which disrupt cell functions. The potential etio-pathological mechanism underlying high glycemic exposure and impaired lung function is chronic low-grade tissue inflammation and microangiopathy of the pulmonary vascular network [36]. Moreover, dysglycemia negatively impairs the lung connective tissue metabolism and causes thickening of the alveolar epithelial and endothelial basement membrane [37,38]. Long-term, low-grade tissue inflammation can contribute to a decline in lung function [39,40]. 

The present study findings also provide insight into the relationship between the HbA1c level, duration of disease, and impaired lung function in T2DM. Moreover, the logistic regression revealed that high HbA1c ≥ 8% was linked with an increased risk for poor lung function and was an indicator of lung damage in a diabetic patient. Thus, our results provide evidence that in diabetic patients, high HbA1c may be an essential clue to find the adverse effect on the lung. 

### Study Strengths and Limitations

There are a few limitations of this study. First, lung function parameters may have been influenced by some other confounding factors. Second, due to cross-sectional design, causality could not be established. Third, we were unable to classify further the diabetic patients based on HbA1c. Despite the few potential limitations, the present study findings are inconsistent with other studies of diabetic patients. The results support the hypothesis that poor glycemic control was related to lung function impairment in individuals with T2DM. Therefore, it is essential to maintain standard glycemic control to minimize lung function impairment in diabetic patients. 

## 5. Conclusions

The findings conclude that high HbA1c or poor glycemic control impairs lung functions in type 2 diabetic patients. Moreover, uncontrolled diabetes mellitus with high HbA1c is more damaging to lung functions compared to the duration of diabetes mellitus. The association between diabetes mellitus and lung function remains essential because of their potential clinical implications. Lung function screening with other routine examinations should be carried out periodically to identify and manage abnormal lung functions in diabetic patients. Normal glycemic control may improve pulmonary functions and the overall health of diabetic patients.

## Figures and Tables

**Table 1:** Demographic and biochemical characteristics of diabetic patients compared to their matched control group.

Parameters	Diabetic Group (*n* = 101)	Control Group (*n* = 101)	*p*-Value
	Mean ± SD	Mean ± SD	
Age (years)	55.50 ± 5.99	54.88 ± 6.76	0.489
Height (cm)	166.02 ± 6.44	166.11 ± 6.04	0.919
Weight (kg)	68.84 ± 4.64	68.21 ± 5.77	0.653
BMI (kg/m^2^)	25.04 ± 2.11	24.34 ± 1.33	0.319
HbA1c (%)	8.19 ± 1.48	6.01 ± 0.24	<0.001 *
Fasting Blood Glucose (mmol/L)	9.12 ± 3.12	5.21 ± 0.11	<0.001 *

* = Significance level.

**Table 2:** Lung function parameters for type 2 diabetic patients with duration of the disease up to five years compared with their matched controls.

Parameters	Diabetic Group (*n* = 14)	Control Group (*n* = 14)	*p*-Value
	Mean ± SD	Mean ± SD	
VC (L)	2.85 ± 0.65	3.09 ± 0.56	0.303
FVC (L)	3.15 ± 0.66	3.47 ± 0.59	0.189
FEV1 (L/Sec)	2.75 ± 0.63	2.98 ± 0.47	0.278
FEV1/FVC Ratio (%)	88.98 ± 6.23	87.65 ± 5.21	0.548
PEFR (L/Sec)	6.88 ± 1.82	7.12 ± 1.67	0.720
FEF-25% (L/Sec)	6.49 ± 1.96	6.77 ± 1.53	0.672
FEF-50% (L/Sec)	4.52 ± 1.79	4.76 ± 1.06	0.907
FEF-75% (L/Sec)	1.71 ± 1.11	1.66 ± 0.69	0.673

* = Significance level.

**Table 3:** Lung function parameters for type 2 diabetic patients with disease duration between 5–10 years compared with their matched controls.

Parameters	Diabetic Group (*n* = 26)	Control Group (*n* = 26)	*p*-Value
	Mean ± SD	Mean ± SD	
VC (L)	2.72 ± 0.80	2.91 ± 0.56	0.328
FVC (L)	3.08 ± 0.91	3.34 ± 0.79	0.283
FEV1 (L/Sec)	2.58 ± 0.75	2.96 ± 0.71	0.068
FEV1/FVC Ratio (%)	86.39 ± 6.79	91.47 ± 5.97	0.006 *
PEFR (L/Sec)	6.55 ± 2.28	7.14 ± 2.15	0.345
FEF-25% (L/Sec)	6.05 ± 2.24	6.83 ± 1.95	0.184
FEF-50% (L/Sec)	3.60 ± 1.29	4.90 ± 1.30	0.001 *
FEF-75% (L/Sec)	1.35 ± 0.61	1.93 ± 0.74	0.003 *

* = Significance level.

**Table 4:** Lung function parameters for type 2 diabetic patients with duration of disease > 10 years compared with their matched controls.

Parameters	Diabetic Group (*n* = 61)	Control Group (*n* = 61)	*p*-Value
	Mean ± SD	Mean ± SD	
VC (L)	2.64 ± 0.60	2.85 ± 0.62	0.055
FVC (L)	3.07 ± 0.75	3.29 ± 0.78	0.106
FEV1 (L/Sec)	2.64 ± 0.61	2.90 ± 0.68	0.029 *
FEV1/FVC Ratio (%)	89.25 ± 7.14	91.35 ± 6.12	0.311
PEFR (L/Sec)	6.56 ± 1.97	7.08 ± 1.94	0.805
FEF-25% (L/Sec)	6.31 ± 1.88	6.80 ± 1.85	0.636
FEF-50% (L/Sec)	4.23 ± 1.26	4.73 ± 1.40	0.483
FEF-75% (L/Sec)	1.62 ± 0.80	1.86 ± 0.74	0.747

* = Significance level.

**Table 5:** Lung functions parameters for type 2 diabetic patients with HbA1c ≤ 8 compared with their matched controls.

Parameters	Diabetic Group (*n* = 58)	Control Group (*n* = 58)	*p*-Value
	Mean ± SD	Mean ± SD	
VC (L)	2.78 ± 0.67	8.94 ± 0.71	0.309
FVC (L)	3.24 ± 0.75	3.34 ± 0.76	0.502
FEV1 (L/Sec)	2.77 ± 0.63	2.95 ± 0.65	0.145
FEV1/FVC Ratio (%)	88.04 ± 6.85	91.32 ± 5.69	0.006 *
PEFR (L/Sec)	6.94 ± 1.86	7.15 ± 1.98	0.542
FEF-25% (L/Sec)	6.58 ± 1.81	6.87 ± 1.84	0.394
FEF-50% (L/Sec)	4.29 ± 1.41	4.85 ± 1.36	0.034 *
FEF-75% (L/Sec)	1.63 ± 0.92	1.95 ± 0.81	0.053

* = Significance level.

**Table 6:** Lung functions parameters for type 2 diabetic patients with HbA1c > 8 compared with their matched controls.

Parameters	Diabetic Group (*n* = 43)	Control Group (*n* = 43)	*p*-Value
	Mean ± SD	Mean ± SD	
VC (L)	2.56 ± 0.64	2.98 ± 0.57	0.002 *
FVC (L)	2.87 ± 0.76	3.39 ± 0.75	0.002 *
FEV1 (L/Sec)	2.46 ± 0.63	2.99 ± 0.65	0.001 *
FEV1/FVC Ratio (%)	89.06 ± 7.11	91.00 ± 5.82	0.175
PEFR (L/Sec)	6.16 ± 2.17	7.20 ± 2.01	0.024 *
FEF-25% (L/Sec)	5.86 ± 2.12	6.96 ± 1.99	0.015 *
FEF-50% (L/Sec)	3.86 ± 1.30	4.92 ± 1.53	0.001 *
FEF-75% (L/Sec)	1.47 ± 0.62	1.94 ± 0.79	0.003 *

* = Significance level.

## Data Availability

Data may be provided on reasonable request to corresponding author.
